# CoWO_4_/Reduced Graphene Oxide Nanocomposite-Modified Screen-Printed Carbon Electrode for Enhanced Voltammetric Determination of 2,4-Dichlorophenol in Water Samples

**DOI:** 10.3390/mi15111360

**Published:** 2024-11-09

**Authors:** Somayeh Tajik, Hadi Beitollahi, Fariba Garkani Nejad, Reza Zaimbashi

**Affiliations:** 1 Research Center of Tropical and Infectious Diseases, Kerman University of Medical Sciences, Kerman 7616913555, Iran; tajik_s1365@yahoo.com; 2 Environment Department, Institute of Science and High Technology and Environmental Sciences, Graduate University of Advanced Technology, Kerman 7631885356, Iran; f.garkani95@gmail.com (F.G.N.); r.zaim2017@gmail.com (R.Z.)

**Keywords:** water pollution, 2,4-dichlorophenol, phenolic compounds, screen-printed carbon electrode, electrochemical sensor

## Abstract

Water pollution with phenolic compounds is a serious environmental issue that can pose a major threat to the water sources. This pollution can come from various agricultural and industrial activities. Phenolic compounds can have detrimental effects on both human health and the environment. Therefore, it is essential to develop and improve analytical methods for determination of these compounds in the water samples. In this work, the aim was to design and develop an electrochemical sensing platform for the determination of 2,4-dichlorophenol (2,4-DCP) in water samples. In this regard, a nanocomposite consisting of CoWO_4_ nanoparticles (NPs) anchored on reduced graphene oxide nanosheets (rGO NSs) was prepared through a facile hydrothermal method. The formation of the CoWO_4_/rGO nanocomposite was confirmed via different characterization techniques. Then, the prepared CoWO_4_/rGO nanocomposite was used to modify the surface of a screen-printed carbon electrode (SPCE) for enhanced determination of 2,4-DCP. The good electrochemical response of the modified SPCE towards the oxidation of 2,4-DCP was observed by using cyclic voltammetry (CV) due to the good properties of CoWO_4_ NPs and rGO NSs along with their synergistic effects. Under optimized conditions, the CoWO_4_/rGO/SPCE sensor demonstrated a broad linear detection range (0.001 to 100.0 µM) and low limit of detection (LOD) (0.0007 µM) for 2,4-DCP determination. Also, the sensitivity of CoWO_4_/rGO/SPCE for detecting 2,4-DCP was 0.3315 µA/µM. In addition, the good recoveries for determining spiked 2,4-DCP in the water samples at the surface of CoWO_4_/rGO/SPCE showed its potential for determination of this compound in real samples.

## 1. Introduction

Environmental pollution, especially contamination of water sources, has involved human societies and other living organisms in serious problems. Along with the increasing population growth, the use of chemicals has also increased in various industrial, domestic, and agricultural fields [1]. The discharge of chemical compounds into water sources is a significant cause of water pollution. Recent studies have shown that phenol and its derivatives are a group of compounds found in environmental samples, so they comprise an important category of environmental pollutants [2]. The presence of phenolic compounds in water samples has attracted considerable interest from environmental protection agencies (EPAs) and scientific institutions due to their high toxicity, low biodegradability, and high tendency to persist in the environment for a long time [3]. Being one of the more toxic phenolic compounds, 2,4-dichlorophenol (2,4-DCP) is extensively utilized in the production of pesticides, insecticides, fungicides, herbicides, pharmaceuticals, preservatives, dyes and etc., leading to significant environmental residues [4]. Due to its highly toxic, poorly biodegradable, and potentially mutagenic and carcinogenic properties, 2,4-DCP has been classified as a priority pollutant by the US EPA [5]. According to the above descriptions, developing a simple, fast, and reliable analytical method for detection of 2,4-DCP in water samples is very important from the aspects of public health and environmental safety. In the recent decades, various analytical methods such as chemiluminescence [6], colorimetry [7], high-performance liquid chromatography (HPLC) [8], capillary electrophoresis [9], gas chromatography–mass spectrometry (GC-MS) [10], and electrochemistry [11,12,13] have been employed to detect 2,4-DCP in various samples.

When comparing these methods, the electrochemical methods are greatly preferred due to their low-cost, simple instruments, simple operation, fast analysis, and sensitive results [14,15,16,17,18,19,20]. Nevertheless, due to the sluggish electron transfer reactions and electrode fouling, the majority of unmodified electrodes require suitable materials for modifications prior to detection [21,22,23,24,25,26,27]. Screen-printed electrodes (SPEs) are typically produced by printing conducting materials such as carbon materials or metal inks on a substrate (usually ceramic) by a screen-printing process. The screen-printing technology allows the mass production of electrodes with favorable characteristics for specific applications. SPEs can be very effective in various fields such as quality control, monitoring and controlling of various processes, quantitative measurements, and more. Also, the SPEs are produced in miniaturized formats, which is very efficient for applications that require small and portable sensors. In addition to this, the miniaturized SPEs can be easily integrated with wearable devices or microfluidic systems, facilitating a broad spectrum of applications in environmental monitoring and medical diagnosis. Notably, improving the performance of SPEs by modifying their surface is an important process in increasing the efficiency of electrochemical sensors based on this type of electrode [28,29,30].

Nanoscience and nanotechnology constitute a growing field of study encompassing structures, devices, and systems characterized by unique properties and functions. Nanotechnology plays a significant role in almost every scientific discipline, such as materials science, physics, chemistry, medicine, engineering, and etc. [31,32,33,34,35,36,37,38,39]. Notably, the use of nanostructures to modify electrodes in electrochemical sensors is one of the basic and interesting applications of this technology. By modifying the electrodes with nanostructures, the electrochemical properties can be improved. These modifications can lead to increased sensitivity, greater selectivity, faster response times, and lower LODs of measurements [40,41,42,43,44,45]. The nanostructures of binary transition metal oxides (BTMOs) show good electrochemical properties due to the synergistic effects between two transition metals and are known as suitable and efficient electrode materials [46,47,48]. According to some scientific reports, CoWO_4_, considered as one of the most crucial compounds, exhibits good catalytic and chemical properties [49,50]. However, several efforts have been made to improve the electrochemical capabilities of BTMOs [51,52]. Developing a method for preparing a nanocomposite of binary transition metal oxides with carbon nanostructures with high conductivity can be considered as an effective way. The combination of carbon nanostructures such as two-dimensional reduced graphene oxide (rGO) with BTMOs can be desirable to enhance the electrical conductivity and electrochemical properties of the resulting nanocomposites due to their high electrical conductivity, large surface area, and good mechanical features [53,54].

In the present study, we aimed to design an electrochemical sensing platform based on CoWO_4_/rGO nanocomposite-modified SPCE for the simple and sensitive determination of 2,4-DCP, which benefits from the advantages of both CoWO_4_ NPs and rGO NSs to improve the performance of SPCEs. To the best of our knowledge, there were no research works reporting the application of CoWO_4_/rGO nanocomposite for SPCE modification to determine 2,4-DCP. The results from this study can help the fabrication of improved electrochemical sensors for 2,4-DCP determination.

## 2. Experimental Section

### 2.1. Materials and Instrumentation

The high-purity solvents and reagents used in this work did not require additional purifications. They were bought from Sigma-Aldrich, Merck, (Darmstadt, Germany).

An electron microscope (field-emission scanning electron microscope—MIRA3 (TESCAN, Brno, Czech Republic)) was used for morphological analysis of the prepared nanocomposite. Also, the XRD pattern of the sample was recorded through an X-ray diffractometer (X’Pert Pro (Panalytical, Almelo, The Netherlands)). All the electrochemical tests to evaluate the performance of the proposed sensor in determining 2,4-DCP in the water samples were carried out using a potentiostat/galvanostat instrument (Autolab/PGSTAT302N (Metrohm, Herisau, Switzerland)). Commercial SPCEs (DS-110 (DropSens, DRP-110, Asturias, Spain) containing three electrodes (1: WE = carbon working electrode, 2: RE = Ag pseudo-reference electrode, and 3: CE = carbon counter electrode) printed on the same planar platform (ceramic platform) were used for performing electrochemical tests.

### 2.2. Synthesis of CoWO_4_/rGO Nanocomposite

The synthesis process of the CoWO_4_/rGO nanocomposite was performed based on the method reported by Xu et al. with some modifications [55]. For this purpose, an aqueous suspension of GO was prepared by dispersing 60 mg of GO into 40 mL of deionized water and ultrasonicating for 1 h. After ultrasonication, aqueous solutions (10 mL containing 2 mmol CoCl_2_.6H_2_O (0.475 g) and 10 mL containing 2 mmol Na_2_WO_4_.2H_2_O (0.659 g)) were added to the above suspension and magnetically stirred for 1 h. Then, the suspension of GO containing metal salts was transferred into a 100 mL Teflon-lined stainless-steel autoclave. After that, it was placed in an oven at 180 °C for 12 h and then naturally cooled to ambient temperature. After collecting the prepared precipitate by centrifugation, the product was obtained after washing and drying (70 °C for 15 h) processes and regarded as CoWO_4_/rGO nanocomposite.

### 2.3. SPCE Modification Using CoWO_4_/rGO Nanocomposite

For the modification process of the SPCE, a homogeneous suspension was firstly prepared by dispersing 1.0 mg of CoWO_4_/rGO nanocomposite into 1.0 mL of deionized water (1 mg/mL suspension). Then, 3.0 µL of the aqueous suspension of CoWO_4_/rGO nanocomposite was dropped on the WE in the SPCE, and the solvent was gradually evaporated under ambient conditions. It was regarded as CoWO_4_/rGO/SPCE.

## 3. Results and Discussion

### 3.1. Characterization of CoWO_4_/rGO Nanocomposite

**Figure 1:**
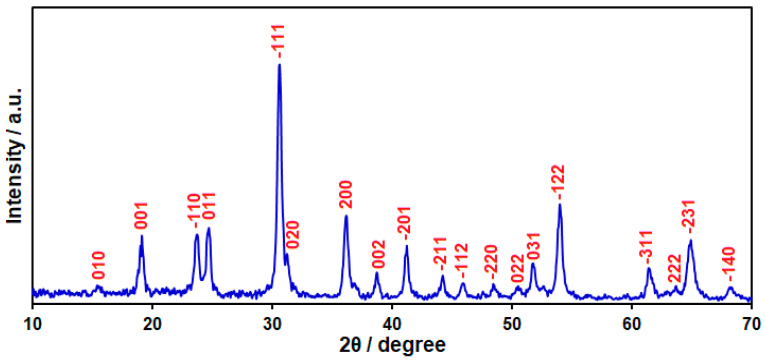
XRD pattern of CoWO_4_/rGO nanocomposite.

The XRD pattern was recorded to clarify the crystalline structure of the prepared nanocomposite, as illustrated in Figure 1. From the XRD pattern, the observed peaks at 15.4, 19.0, 23.6, 24.7, 30.5, 31.1, 36.0, 38.6, 41.1, 44.1, 45.8, 48.4, 50.4, 51.7, 54.0, 61.3, 63.6, 64.8, and 68.1 are related to the diffraction of the (010), (001), (−110), (011), (−111), (020), (200), (002), (−201), (−211), (−112), (−220), (022), (031), (−122), (−311), (222), (−231), and (−140) planes of CoWO_4_, respectively (JCPDS No. 15-0867) [56,57]. Nonetheless, the diffraction peaks of rGO were not observed in the XRD pattern of the CoWO_4_/rGO nanocomposite. The diffraction peaks of rGO are probably covered by the sharp diffraction peaks of CoWO_4_ NPs.

**Figure 2:**
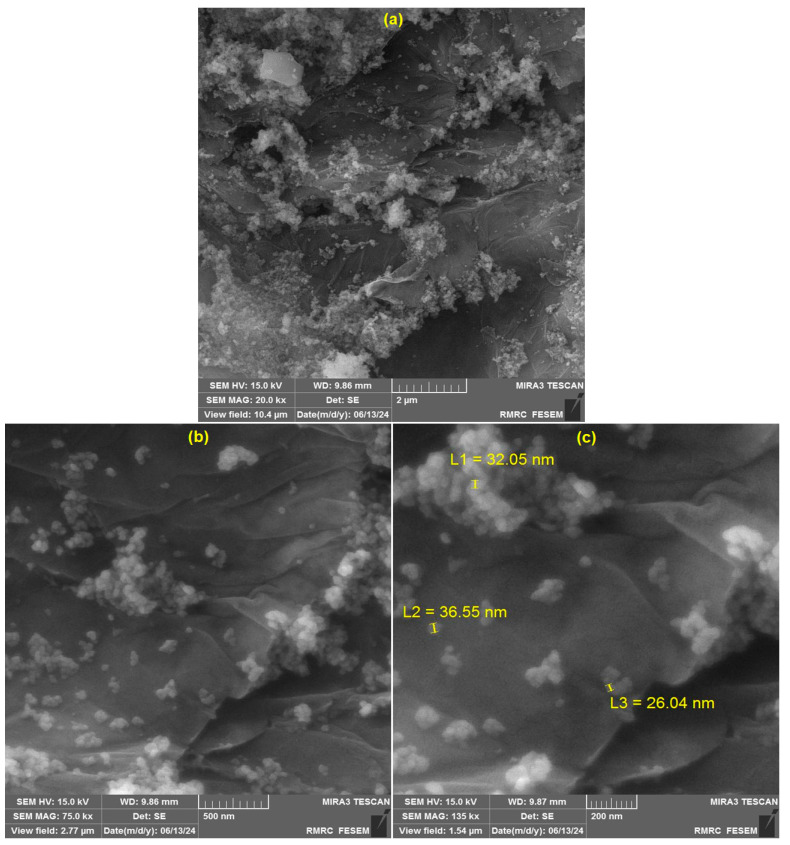
FE-SEM images of CoWO_4_/rGO nanocomposite at (**a**) 2 µm, (**b**) 500 nm, and (**c**) 200 nm magnifications.

The surface morphology of the as-prepared CoWO_4_/rGO nanocomposite was observed by FE-SEM images. As shown in FE-SEM images at different magnifications (Figure 2), the rGO nanosheets were decorated with small nanoparticles of CoWO_4_. Also, the FE-SEM images show the aggregation of semispherical nanoparticles of CoWO_4_.

### 3.2. Electrochemical Response of CoWO_4_/rGO Modified SPCE Compared to Unmodified SPCE as Electrochemical Sensors for 2,4-DCP Determination

The pH of the buffer solution is considered to be a significant parameter that can affect the performance of sensors for electroanalysis of compounds. Therefore, the effect of the pH of phosphate buffer solution (PBS 0.1 M) in the range 3.0 to 9.0 on the responses of CoWO_4_/rGO/SPCE for 2,4-DCP oxidation (30.0 µM) was assessed. The differential pulse voltammetry (DPV) analyses demonstrated that the anodic peak currents (Ipa) of 2,4-DCP when increasing the pH from 3.0 to 7.0 reached the maximum value at pH 7.0, and then decreased beyond pH 7.0. Based on the observed maximum value of Ipa at pH 7.0, other electrochemical studies and measurements were conducted in 0.1 M PBS with pH 7.0.

**Figure 3:**
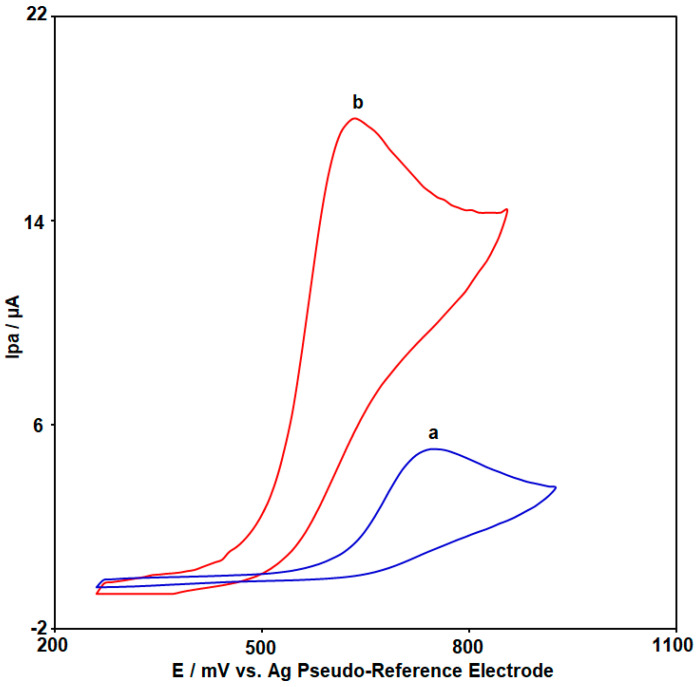
CV responses of unmodified SPCE: cyclic voltammogram (a) and CoWO_4_/rGO nanocomposite-modified SPCE: cyclic voltammogram (b) in buffer solution containing 50.0 µM 2,4-DCP (scan rate (υ): 50 mV/s).

For comparison of the sensing performance of an unmodified SPCE and the CoWO_4_/rGO/SPCE, cyclic voltammograms were recorded in buffer solution containing 50.0 µM 2,4-DCP, which are illustrated in Figure 3. The response of the unmodified SPCE towards the oxidation of 2,4-DCP was poor, where the oxidation peak was observed at 750 mV with Ipa of 5.1 µA (cyclic voltammogram a). In contrast, an obvious oxidation peak of 2,4-DCP was detected with the CoWO_4_/rGO/SPCE (cyclic voltammogram b). By modifying the surface of the SPCE, along with the significant increase in the Ipa of 2,4-DCP (17.9 µA), the anodic peak potential (Epa) was also decreased (640 mV). The significant enhancement of the electrochemical response was related to the considerable synergistic effects of CoWO_4_ NPs and rGO NSs in the oxidation process of 2,4-DCP.

### 3.3. Effect of Scan Rate on the Electrochemical Response of CoWO_4_/rGO/SPCE Towards the Oxidation of 2,4-DCP

**Figure 4:**
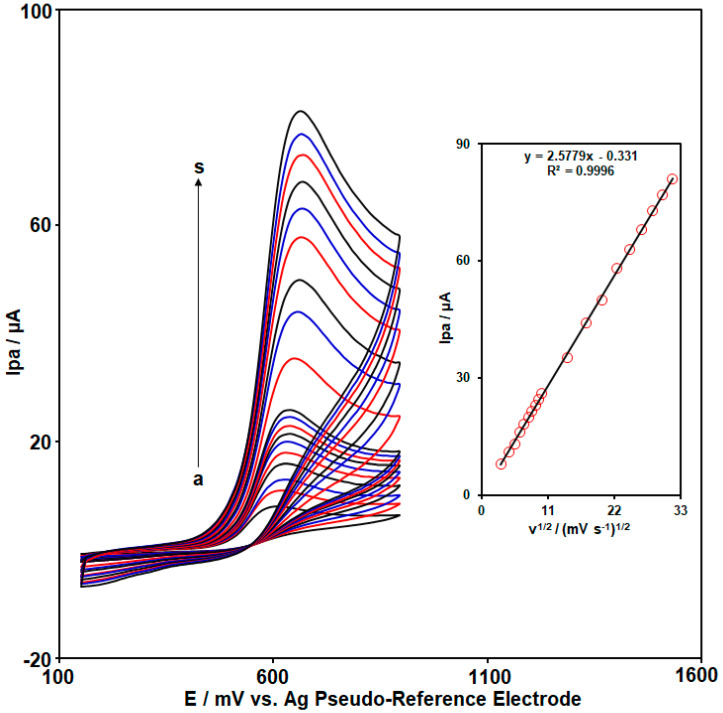
CV responses of CoWO_4_/rGO/SPCE in the buffer solution containing 50.0 µM 2,4-DCP at various scan rates (cyclic voltammograms of (a) to (s) are as follows: (a) υ: 10, (b) υ: 20, (c) υ: 30, (d) υ: 40, (e) υ: 50, (f) υ: 60, (g) υ: 70, (h) υ: 80, (i) υ: 90, (j) υ: 100, (k) υ: 200, (l) υ: 300, (m) υ: 400, (n) υ: 500, (o) υ: 600, (p) υ: 700, (q) υ: 800, (r) υ: 900, and (s) υ: 1000 mV/s). Inset: Linear dependence between Ipa and υ^1/2^.

CV analysis at various scan rates (from υ: 10 mV/s to υ: 1000 mV/s) was also used to assess the response of CoWO_4_/rGO/SPCE to oxidation of 50.0 µM 2,4-DCP in buffer solution (Figure 4). According to the CVs shown in Figure 4, the current intensity of oxidation peaks increased continuously with the increase in scan rate. Figure 4 (Inset) exhibits the suitable linearity between Ipa and υ^1/2^ (Ipa (µA) = 2.5779υ^1/2^ (mV/s)^1/2^–0.331) (R^2^ = 0.9996), which revealed that the electro-oxidation of 2,4-DCP on the CoWO_4_/rGO/SPCE was under the diffusion-controlled process. Moreover, a slight shift in the Epa of 2,4-DCP toward the positive side was observed, which can be related to slower electron transfer at faster scan rates.

### 3.4. Chronoamperometric Investigations of 2,4-DCP at CoWO_4_/rGO/SPCE

**Figure 5:**
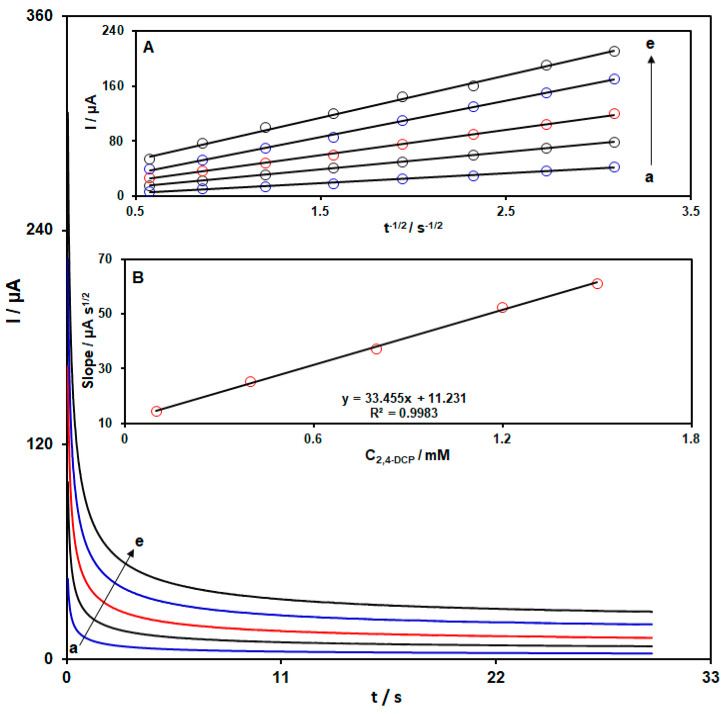
Chronoamperograms for the oxidation of 2,4-DCP concentrations on the CoWO_4_/rGO/SPCE in buffer solution (chronoamperograms (a) to (e) are related to (a) C: 0.1 mM, (b) C: 0.4 mM, (c) C: 0.8 mM, (d) C: 1.2 mM, and (e) C: 1.5 mM). Inset (**A**): Plots of I versus t^−1/2^ obtained from chronoamperograms; inset (**B**): Plot of the slope of the straight lines versus the 2,4-DCP concentration.

The oxidation of 2,4-DCP on the CoWO_4_/rGO/SPCE was further investigated using chronoamperometry. The chronoamperograms shown in Figure 5 were recorded for different concentrations of 2,4-DCP in the buffer solution on the CoWO_4_/rGO/SPCE by applying a step potential of 700 mV. Based on the recorded chronoamperograms, an increase in the concentration of 2,4-DCP was associated with an increase in the anodic currents. In the chronoamperometric investigations, it is possible to measure the diffusion coefficient (D) of electroactive compounds on the basis of the Cottrell equation: I = nFACD^1/2^π^−1/2^t^−1/2^. The Cottrell curves (I-t^−1/2^ curves) demonstrated a linear relationship over a certain range of time (Figure 5A). The Cottrell curves were plotted using chronoamperograms recorded for a given concentration of 2,4-DCP. Then, the slope of the Cottrell curves was plotted against the different concentrations of 2,4-DCP (Figure 5B). Finally, from the slope of the resulting plot in Figure 5B and using the Cottrell’s equation, the D parameter was found to be 9.5 × 10^−5^ cm^2^/s for 2,4-DCP.

### 3.5. Electroanalysis Performance of CoWO_4_/rGO/SPCE for 2,4-DCP

**Figure 6:**
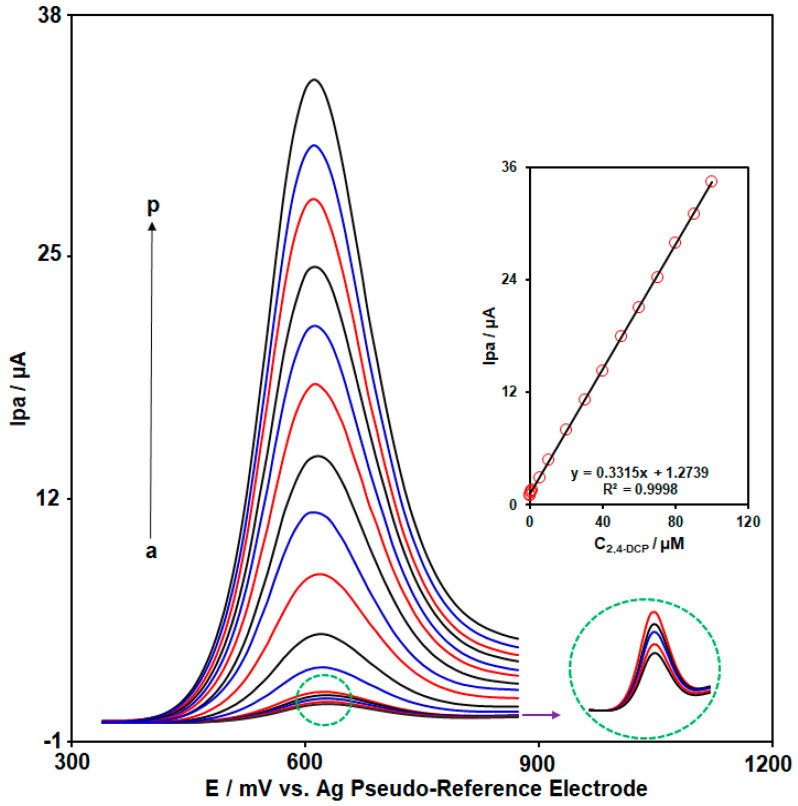
DPV responses of CoWO_4_/rGO/SPCE to various concentrations of 2,4-DCP in the buffer solution (DPVs of (a) to (p) indicate the following: (a) C: 0.001, (b) C: 0.01, (c) C: 0.1, (d) C: 0.5, (e) C: 1.0, (f) C: 5.0, (g) C: 10.0, (h) C: 20.0, (i) C: 30.0, (j) C: 40.0, (k) C: 50.0, (l) C: 60.0, (m) C: 70.0, (n) C: 80.0, (o) C: 90.0, and (p) C: 100.0 µM). Inset: calibration plot of 2,4-DCP.

DPV is a highly versatile method for the detection of trace amounts of species. In the DPV technique, constant-amplitude potential pulses are applied to a working electrode. In this technique, the current is measured at two points for each pulse (1: before the pulse application and 2: at the end of the pulse). The difference between two measurements helps to isolate and quantify the current associated specifically with the electrochemical process, while minimizing the contribution from background currents. DPV is usually known for its high sensitivity, enhanced peak resolution, lower detection limits, and reduced background current compared to methods like CV. Therefore, DPV technique was applied for quantitative analysis of 2,4-DCP on the CoWO_4_/rGO/SPCE. The DPV responses of CoWO_4_/rGO/SPCE to different concentrations of 2,4-DCP in the buffer solution are provided in Figure 6. As the 2,4-DCP concentration increased, there was a linear relationship between the Ipa and 2,4-DCP concentration (linear range: 0.001 µM to 100.0 µM) (inset of Figure 6). Also, the calculated LOD of the CoWO_4_/rGO/SPCE for 2,4-DCP was 0.0007 µM. Table 1 demonstrates the comparison between the sensor developed in our work and some other electrochemical sensors reported by various research groups for 2,4-DCP determination. Compared to some reported electrochemical sensors for determination of 2,4-DCP, as shown in the Table 1, the electrochemical sensor based on a SPCE modified with CoWO_4_/rGO nanocomposite has the advantages of lower LOD and wider linear range. Also, the efficiency of this sensor is comparable to others. Therefore, it can be concluded that the good performance of the CoWO_4_/rGO/SPCE sensor for 2,4-DCP determination is related to the synergistic effects of rGO nanosheets and CoWO_4_ NPs.

### 3.6. Stability, Reproducibility, and Repeatability Studies of CoWO_4_/rGO Modified SPCE

Firstly, the CoWO_4_/rGO/SPCE was stored in ambient temperature for 15 days and the response current of this sensor towards 40.0 µM 2,4-DCP in buffer solution was recorded every three days to evaluate its stability. After 15 days, it was observed that the decrease in the peak current was 4.7% compared to that of the initial response current of the sensor. In order to investigating the reproducibility, the DPV method was applied to record the response current of five modified SPCEs in detecting a buffer solution containing 40.0 µM 2,4-DCP. The relative standard deviation (RSD) from the current responses of these electrodes was calculated to be less than 4%. Eventually, the repeatability of the response of the CoWO_4_/rGO/SPCE was assessed by performing 10 successive voltammetric (DPV) measurements of 40.0 µM 2,4-DCP in the buffer solution. After 10 successive measurements of 2,4-DCP, 93.8% of the initial response current was retained. The findings from these studies revealed that the CoWO_4_/rGO/SPCE sensor had good stability, reproducibility, and repeatability.

### 3.7. Effects of Interfering Species

An important challenge for the applicability of a designed sensor is its performance to detect a target analyte in the presence of coexisting species in real samples. Based on this, the electrochemical response of the CoWO_4_/rGO/SPCE sensor towards 2,4-DCP (20.0 µM) was compared in the absence and presence of interfering species (some ions and phenolic compounds). The results based on DPV measurements demonstrate that the presence of 200-fold concentrations of Mg^2+^, Na^+^, Ca^2+^, Li^+^, NH_4_^+^, Cl^−^, SO_4_^2−^, and NO_3_^−^ and a 50-fold concentration of hydroquinone had almost no significant influence on the determination of 2,4-DCP, with signal changes below 5%.

### 3.8. Real Sample Analysis

Finally, to investigate the practical application of CoWO_4_/rGO/SPCE, the standard addition method was employed to determine 2,4-DCP in tap water and well water samples. The water samples were collected, filtered, and utilized as real samples. To perform this investigation, the filtrated water samples were diluted with buffer solution. Then, standard solutions of 2,4-DCP with various concentrations were added to the water samples, and the DPVs were recorded with the CoWO_4_/rGO/SPCE to determine the content of 2,4-DCP in these samples. The obtained results are provided in Table 2. As can be seen, the recoveries of 2,4-DCP in the water samples were 97.3% to 104.3% after adding standard solutions. Also, the relative standard deviations (RSDs) ranged from 1.9% to 3.4%. These results demonstrate that the developed sensing platform was suitable for detecting 2,4-DCP in water samples.

## 4. Conclusions

In summary, the electrochemical determination of 2,4-DCP using a CoWO_4_/rGO nanocomposite-modified SPCE was successfully carried out in this work. This nanocomposite was prepared by the hydrothermal method and its characterization was conducted by XRD and FE-SEM methods. Considering the synergistic effects of CoWO_4_ NPs and rGO NSs, the CoWO_4_/rGO nanocomposite-modified SPCE demonstrated improved performance for oxidation of 2,4-DCP compared to the unmodified SPCE. The results from quantitative measurements by the DPV technique indicated a wide linear range of 0.001 to 100.0 µM and a low LOD of 0.0007 µM for 2,4-DCP determination. Also, the studies performed to assess the stability, reproducibility, and repeatability features of the developed sensor showed acceptable results. Finally, measurements in real water samples were successfully conducted using the CoWO_4_/rGO/SPCE sensor with an excellent recovery of 97.3% to 104.3%. Therefore, the proposed sensor can present good performance for its utilization in detecting 2,4-DCP in the water samples with the purpose to control environmental pollution.

## Figures and Tables

**Table 1:** Comparison the values of LOD and linear range of the CoWO_4_/rGO/SPCE sensor with some of the previously published reports.

Modified Electrode	Detection Method	Linear Range	LOD	Reference
Carbon dots–hexadecyltrimethyl ammonium bromide–chitosan (CDs-CTAB-CS) modified glassy carbon electrode (GCE)	DPV	0.04 µM to 8 µM	0.01 µM	[5]
Cu-based metal–organic framework/electrochemically reduced graphene oxide composite (Cu-MOF/ErGO) modified GCE	DPV	1.5 µM to 24 µM	0.083 µM	[11]
Au nanoflakes/ZrO_2_ nanocomposite modified GCE	DPV	1.5 µM to 24 µM	0.053 µM	[12]
Rutin-rGO-TiO_2_ nanocomposite modified GCE	DPV	5 µM to 150 µM	0.02 µM	[58]
Diamond–graphene–polyaniline modified GCE	Square wave voltammetry (SWV)	5 µM to 80 µM	0.25 µM	[59]
Molybdenum disulfide-ionic liquid-Au-Ag nanorods (MoS_2_-IL-Au-Ag NRs) modified GCE	DPV	0.01 µM to 50 µM	2.6 nM	[60]
β-cyclodextrin functionalized IL modified carbon paste electrode (CPE)	Amperometry	4 µM to 100 µM	1.2 µM	[61]
CoWO_4_/rGO modified SPCE	DPV	0.001 µM to 100.0 µM	0.0007 µM	This work

**Table 2:** Results from the analysis of water samples at CoWO_4_/rGO/SPCE.

Sample	Concentration of 2,4-DCP (µM)		Results	
	Added	Found	Recovery (%)	R.S.D. (%)
Tap water	0	-	-	-
	5.0	4.9	98.0	3.4
	7.0	7.3	104.3	1.9
	9.0	9.1	101.1	2.7
	11.0	10.7	97.3	2.2
Well water	0	-	-	-
	5.5	5.6	101.8	2.5
	7.5	7.4	98.7	3.0
	9.5	9.8	103.2	2.3
	11.5	11.4	99.1	2.8

## Data Availability

The data presented in this study are available on request from the corresponding authors.
